# Managing a Pan-European Consortium on Late Effects among Long-Term Survivors of Childhood and Adolescent Cancer—The PanCareLIFE Project

**DOI:** 10.3390/ijerph18083918

**Published:** 2021-04-08

**Authors:** Peter Kaatsch, Julianne Byrne, Desiree Grabow, on behalf of the PanCareLIFE Consortium

**Affiliations:** 1 German Childhood Cancer Registry (GCCR), Institute of Medical Biostatistics, Epidemiology and Informatics, University Medical Center, 55131 Mainz, Germany; 2 Boyne Research Institute, 5 Bolton Square, East, Drogheda, A92 RY6K Co. Louth, Ireland; jmmbyrne925@gmail.com; 3 German Childhood Cancer Registry, Division of Childhood Cancer Epidemiology, Institute of Medical Biostatistics, Epidemiology and Informatics (IMBEI), Johannes-Gutenberg University, 55131 Mainz, Germany; desiree.grabow@uni-mainz.de

**Keywords:** late effects, child, cancer survivor, health care, epidemiology, fertility, hearing loss, health-related quality of life

## Abstract

PanCareLIFE brought together European partners and is the largest study to have evaluated the issues of fertility impairment, hearing loss, and health-related quality of life in survivors of childhood and adolescent cancer. Successful delivery of the project aims did not evolve solely from scientific qualities. Organizational structure and careful information management were key components for its successful completion and are retrospectively assessed in this paper. PanCareLIFE used cohort studies, case-control studies, clinical evaluation of hearing, and genetic testing to study 32,000 survivors from 25 data providers. A management team implemented the organizational structures, was the decision making body, developed and maintained a communication plan, and supervised deadlines, and made timely decisions. A biostatistics support group and an ethical advisory board were established. A publication committee ensured quality and accuracy of publications and is jointly responsible for the sustainability of the project. The chosen management structure of PanCareLIFE can serve as a blueprint for the management of complex international projects. Apart from the survivors themselves, various target audiences like oncology researchers, health care providers, and policy makers can derive benefits from the project. The results can also be used in oncological frontline therapy to reduce toxicity.

## 1. Introduction

A consequence of increasing survival probabilities after childhood cancer is the growing number of childhood cancer survivors. Estimates show that approximately 1 in every 640 young adults is a survivor of childhood cancer [1]. However, the treatment regimens are harsh and their long-term complications can be serious. Over one-quarter of childhood cancer survivors have at least one severe or life-threatening adverse event [2,3,4] such as a second malignancy (e.g., in Nordic countries [5], in Germany [6], in a pan-European project [7,8,9], or cardiac disease [10,11,12]) with markedly increased risks persisting throughout life. Furthermore, perhaps as many as 75% have at least one late effect [13], including non-life-threatening late effects [14,15]. Compared to the general population, survivors spend on average five times as many days in hospital and face various life challenges [16,17,18].

To achieve statistically significant and clinically meaningful results, studies of late effects require the recruitment of large survivor cohorts, which can only be derived from international collaboration [19]. PanCareLIFE comprises an unprecedented series of European survivors of childhood, adolescent, and young adult cancer (totaling more than 32,000) derived from institutions in 12 countries and from two international clinical trial groups. It assembled a team of European experts in the fields of epidemiology, clinical medicine, pediatric oncology, endocrinology, audiology, ethics, and genetics to address risk assessment for these survivors.

Managing late effects is essential to providing the best possible long-term care after cancer is cured. As the number of survivors increases, the treatment of late effects will place an additional burden on health care systems. Research-derived data can be used to prevent or alleviate the impact and occurrence of late effects by developing models that match individual patients with effective treatments having the least late effects. They can be used to develop evidence-based guidelines, which form the basis of personalized counselling and education. In the long run these strategies will minimize the occurrence and impact of late effects on survivors and their families.

PanCareLIFE addresses three specific issues in long-term survival that can seriously impact survivors: fertility, hearing impairment, and health-related quality of life. The phrase “long-term” generally relates to survivors who have survived at least five years. PanCareLIFE was conceived to give more insights into genetic susceptibility and to prevent or reduce the impact of these late effects on cancer survivors. A companion paper describes the scientific basis for the PanCareLIFE consortium [20]. This paper focuses on assessing the organizational structure from a retrospective view point and provides an outlook on possibilities for ensuring the sustainability of such collaborative projects in the future.

## 2. Materials and Methods

### 2.1. Aims

The PanCare network, which was established in 2008, is a multidisciplinary pan-European network of professionals, survivors, and their families that aims to reduce the frequency, severity, and impact of late side-effects of the treatment of children and adolescents with cancer [21]. The PanCareLIFE consortium originated from this network and addresses late effects that potentially reduce the quality of life of survivors, namely hearing and fertility impairments, as well as health-related quality of life. PanCareLIFE started in November 2013 and ended in October 2018. Summarized information on the objectives and aims of the project are provided by the Community Research and Development Information Service (CORDIS) of the European Commission [22].

Survivors with a cancer diagnosis detected at an age below 25 years were included in PanCareLIFE. Within the specific work packages more detailed inclusion and exclusion criteria were implemented.

The project evaluated the risks of the aforementioned late effects, so that healthcare providers can stratify survivors into risk categories by using genetic information combined with data on treatment and other factors. This will enable personalized, evidence-based long-term care and the best health-related quality of life for each survivor. It has also developed evidence-based guidelines for male and female fertility preservation. PanCareLIFE collected data on about 32,000 well-characterized childhood cancer survivors. From these, for the fertility part of the project more than 6000 survivors completed questionnaires, about 1500 provided serum samples, and more than 400 case-control triplets were included. For the ototoxicity study about 2000 survivors contributed audiograms. More than 10,000 survivors completed questionnaires regarding quality of life. Almost 1000 samples were available for genetic analyses related to ototoxicity and gonadal impairment. These approaches will support the overall aim of improving the health and quality of life of survivors of cancer diagnosed at a young age.

### 2.2. Cooperating Institutions, Data Providers, Work Packages, and Central Data Management

Table 1 shows the co-operating partners involved. A total of 28 beneficiaries and data providers from 13 countries (Austria, Czech Republic, Denmark, France, Germany, Ireland, Israel, Italy, Norway, Poland, Switzerland, The Netherlands, and UK) were involved in PanCareLIFE. Seventeen of these 28 institutions were official partners (“beneficiaries”), who received money directly from the EU, while 11 were data providers, but not beneficiaries (“third parties”). Other co-operating partners were an audiological center, laboratories, and the members of PanCareLIFE’s ethics advisory board. The experts in the cooperating institutions covered a range of cancer research and clinical disciplines, but all had experience in working on multidisciplinary, international projects. Through the collaborative process of generating the proposal and writing the study protocols all partners developed a clear understanding of their roles and responsibilities within the project.

PanCareLIFE consists of eight work packages (WPs). The five scientific work packages were previously described in detail [20]: WP2 Fertility Preservation Guidelines (WP leader: L. Kremer), WP3 Female Fertility Impairment (E. van Dulmen-den Broeder), WP4 Genetics of Fertility Impairment and Hearing Loss (M.M. van der Heuvel-Eibrink), WP5 Ototoxicity (T. Langer), and WP6 Health related Quality of Life (G. Calaminus).

There are two accompanying WPs: WP7 Dissemination and Exploitation (K. O’Brien) and WP8 Project Management (P. Kaatsch). WP7 represents 4% of the whole project budget while project management represents 3%. The remaining 93% were reserved for the five scientific work packages, WP2—WP6, and WP1.

**Figure 1:**
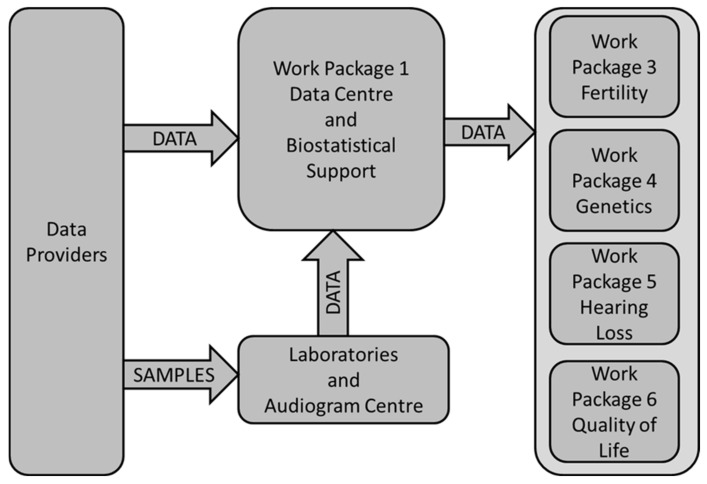
Data flow within the PanCareLIFE project and the central role of the data center in work package 1.

The work package WP1 (Data Centre and Biostatistical Support; P. Kaatsch) was responsible for data management. Its central role is shown in Figure 1: data providers sent data directly to the data center according to variable lists provided by the scientific WPs. Samples (saliva or blood) and audiograms were sent to the laboratories and audiological reference center, respectively. Data generated at these places were additionally sent to the WP1’s central data base. WP1 performed data harmonization (e.g., clarification of uncertainties with data providers arising from plausibility checks); linked the relevant demographic, clinical, genetic, and audiological data; and finally sent plausibility checked data to the scientific WPs for analysis. To consider data protection rules all data were sent in pseudo-anonymized formats.

Regarding ethical approvals and consent to participate, ethical approvals for their respective contributions were obtained by all data providers and all work package leaders in their respective countries or institutions. Each data provider obtained informed consent locally from all participants. All participating institutions provided their own consent forms and ethical approvals to the management.

### 2.3. Management Team, General Assembly, and Executive Board

During the five-year project period several deliverables to the European Commission became due. For example, the deliverables related to project management comprised mainly ethical approval reports (due after 8, 16, and 36 months), as well as meeting minutes. Related to dissemination and exploitation, a deliverables website was set up at the beginning of the project, as well as a joint action plan and a final report on dissemination events toward the project’s end.

While WP leaders were responsible for ensuring that the WP team met the objectives and milestones of their WPs and produced timely deliverables of a high standard, responsibility for the overall management of the project resided with the management team. This was directed by the project coordinator’s team and included the research manager and the administrative manager. The research manager worked closely with the coordinator to monitor progress, to ensure that the project met all contractual deadlines, and delivered all the project objectives. The research manager’s team provided a template for the study protocol and worked with each WP leader to complete the study protocol. The administrative manager provided administrative support, which included contract monitoring, deliverable scheduling, quality assurance and submission of deliverables, coordination of 6-monthly internal reports, minutes from board meetings and general assemblies, periodic reports to the EU, and budgetary monitoring based on partner budget reports. The management team reviewed all deliverables to the EU to ensure all relevant information was provided in a clear and concise manner. The coordinator and research management teams met face-to-face in Mainz several times a year during the five-year course of the project.

Oversight of the project was the responsibility of the general assembly (GA), chaired by the coordinator. Each beneficiary nominated a representative to the GA. The GA ensured that the project proceeded according to plan and that the research met the project objectives. The GA met twice a year, received reports from the management, and had the following tasks: to monitor work progress on a regular basis; supervise the progress and assess the quality of the work; solve any current or upcoming problems; handle intellectual property issues; supervise dissemination of project results; and supervise the overall safety, quality control, gender, and ethical aspects of PanCareLIFE.

**Figure 2:**
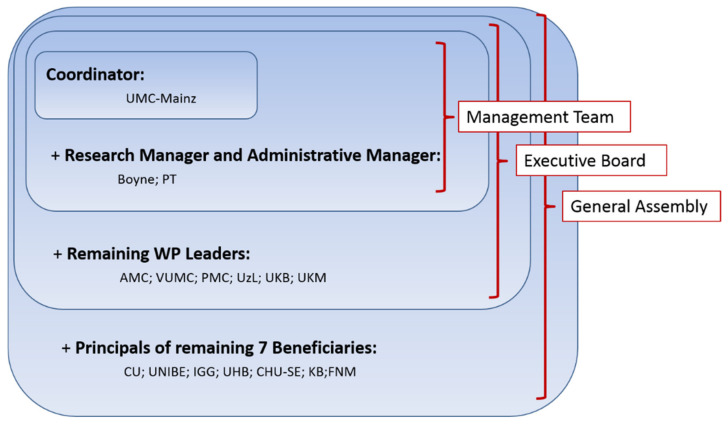
Organizational structure chosen in the PanCareLIFE project (short names stand for beneficiaries briefly explained as follows (city and country) and described in detail in Appendix A under PanCareLIFE project partners): UMC-Mainz: Mainz, Germany; Boyne: Drogheda, Ireland; PT: Dublin, Ireland; AMC: Amsterdam, Netherlands; VUMC: Amsterdam, Netherlands; PMC: Utrecht, Netherlands; UzL: Lübeck, Germany; UKB: Bonn, Germany; UKM: Münster, Germany; CU: Berlin, Germany; UNIBE: Bern, Switzerland; IGG: Genoa, Italy; UHB: Brno, Czech Republic; CHU-SE: Saint Etienne, France; KB: Copenhagen, Denmark; FNM: Prague, Czech Republic.

The executive board, consisting of the management team and the WP leaders, met every two months and was accountable to the GA. The executive board was responsible for the proper execution and implementation of the decisions of the GA. Figure 2 shows the organizational structure of PanCareLIFE.

### 2.4. Advisory and Supporting Groups

Given the retrospective nature of the project and in particular the genetic data and data that were collected when patients were children, PanCareLIFE established an independent ethics advisory board. Its objective was to provide ethical oversight of the consortium and to address ethical issues, such as ethical conduct and liability, biobanking of human samples, storage of data, and management of incidental findings. The board helped with ethical, confidentiality, and informed consent issues, and assisted with the ethical aspects of fertility preservation guidelines.

A biostatistical support group, included in WP1, was established to ensure a uniform approach to PanCareLIFE data, and to support those work packages that needed and requested assistance. The objective was to ensure the high standard of the research and the quality of the resulting publications. The group evaluated each study protocol, gave advice on statistical power issues and other concerns, helped with the statistical analysis plan for each WP, and provided assistance as needed in the analysis and report writing phases. The group also provided technical assistance in exchanging identification numbers of genetic datasets to maintain pseudo-anonymity.

The publication committee was another important and on-going tool with responsibility for managing the publications and presentations arising from PanCareLIFE during and after the project lifetime, to ensure their quality and accuracy, and to monitor potential overlap. The committee implemented a publication policy, setting out guidelines for authorship and procedures for submission of applications of intent for publications and presentations, as well as maintaining a publications database to capture the progress of publications. The committee works closely with the management team to ensure that applications of intent are clearly and concisely written, decide questions of authorship, and review papers before submission. This is also the basis of ensuring the sustainability of the project in the long run.

### 2.5. Communication Strategy and Dissemination Plan

Good communication is the key to ensure the smooth running of any consortium. Clear communication of discussions, decisions, and actions was essential for all members of the consortium to conduct their work in a uniform manner and to avoid confusion. The management team established clear communication channels, a newsletter, and virtual meetings to facilitate open exchange and effective problem solving, including regular telephone conferences: between the management team and WP leaders and data providers, within the management team, and within the executive board. These virtual meetings were supplemented with twice-yearly GA meetings, face-to-face meetings at WP-level, and site visits, where WP leaders or their representatives visited data providers to help to deal with problems. Together, this range of communication mechanisms was essential for creating an effective and efficient working atmosphere. In particular, the research manager attended as many of these meetings as possible to ensure integration of activities across the project. A document sharing facility was also established for the PanCareLIFE consortium for the storage of updated and previous versions of relevant documents. An email list of “members”, comprising more than 100 people, was used for announcements to the entire consortium.

An important part of the work of PanCareLIFE is the dissemination of methods and results to all possible relevant audiences. These include clinicians, cancer researchers, cancer survivors and their families, the general public, patient advocacy and support groups, healthcare providers and policy makers, sister projects, and the media. WP7 has developed a dissemination and exploitation plan that includes a project website (www.pancarelife.eu (accessed on 1 April 2021)) with details of the WPs, meetings, announcements, publications, and other matters of interest to the research community. Social media posts and updates on Facebook and Twitter are a regular feature. Promotional materials, such as brochures and banners, have also been prepared, and translated and printed in different European languages.

## 3. Results

PanCareLIFE collected data on about 32,000 well-characterized childhood cancer survivors from clinical trials and interventions, surveys, hospital-based series, national networks, and cancer registries; including demographic information, treatment data, biospecimens, genetic data, and audiometric data. A total of 25 data providers (Table 1) were included in the PanCareLIFE contributing data. Some of the data providers delivered data addressing only one of the three issues in focus, i.e., fertility, hearing impairment, or health-related quality of life, while others addressed several. The number of research subjects varied per data provider, from several dozen to several thousands.

The management structure of PanCareLIFE was a key component for completing the project on time. The consortium was large and the research topics were diverse. The experience and expertise of each member of the management team was critical to the delivery of the project given the number of data sources included, the different types of analyses, and different types of data to be collected and harmonized. The team worked together with a clear understanding of duties, without overlap, and yet had sufficient redundancy built in, so that work could be managed by others as individuals became indisposed or otherwise on leave. The successful teamwork within our consortium was also expressed by the associated data providers, who were initially not part of the EU application and were therefore not funded by the EU. They regarded the data assembly in this project as attractive in itself. On a working level there was no difference for data providers in being an EU-beneficiary or not, only the attending of meetings was sometimes a bit harder for those not being funded by the EU. Another key component to the success of the PanCareLIFE consortium was the strong sense of working together towards common goals, an understanding of each partner’s strengths and weaknesses, and the good humor that has been maintained throughout the course of the project.

**Figure 3:**
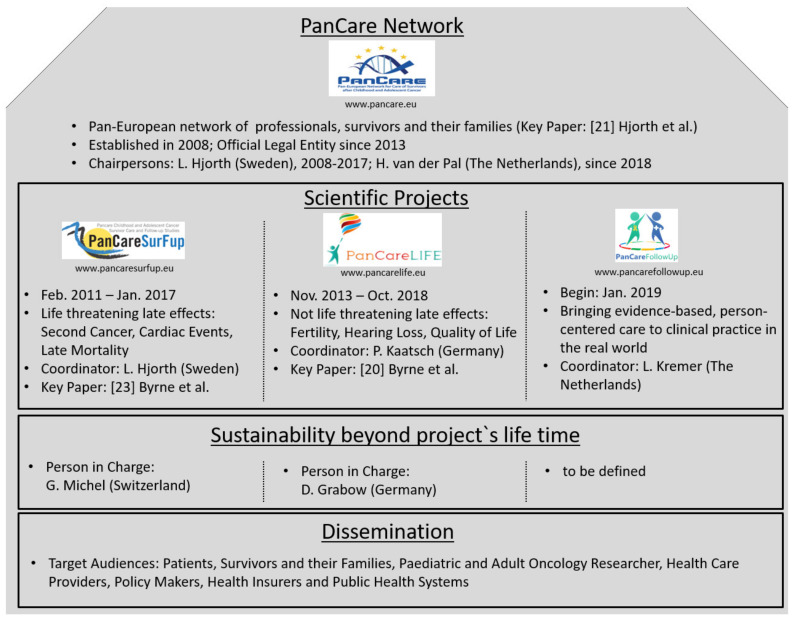
The PanCare network and its EU funded projects PanCareSurFup, PanCareLIFE, and PanCareFollowUp.

PanCareLIFE is one of three EU funded projects built on the work of the PanCare network dealing with the late effects of cancer treatment at a young age. The other two are PanCareSurFup [23] and PanCareFollowUp. An overview about these projects is shown in Figure 3. The close collaboration within PanCare is of great value for this pan-European approach to cancer survivor research. The sustainability of at least the two completed PanCare projects is already ensured. This means that working together beyond the projects’ end and the provision of study records and data for future researchers is ensured. Dissemination is done in manifold ways, for example, by project websites, social media posts, and brochures. The biannual Pancare network meetings, which include representatives of survivor groups, also contribute to the dissemination of new insights.

The increase in knowledge of their own risks for late effects will help the promotion of health among survivors and their families. Adult survivors are becoming better advocates for their own health care. Therefore, PanCareLIFE studies on fertility (WP3, WP4) will contribute information needed to establish specific risk profiles, e.g., for gonadal impairment, as a result of specific therapies, including the impact of chemotherapeutic agents and radiotherapy, as well as genetic variation. The implementation of fertility preservation guidelines (WP2) generated during PanCareLIFE could have a profound impact on long-term outcomes, especially as patients enter adult life. The materials developed and evaluated in the intervention study can be widely used to improve fertility preservation. Results from the ototoxicity study (WP4, WP5), which aimed to describe the occurrence of damage to the inner ear and to investigate genetic susceptibility to hearing impairment, will help to quantify and assess the impact of issues such as platinum-induced toxicity [15]. The PanCareLIFE health-related quality of life study (WP6) will evaluate quality of life in general across national borders, as well as in relation to fertility loss and ototoxicity. Thus, PanCareLIFE will help to quantify the toll of cancer treatment in relation to specific deficits. Some results were published at virtually the same time as the project ended (e.g., [24,25,26]).

## 4. Discussion

The PanCareLIFE consortium expects a number of positive outcomes to result from the project. For instance, the results will include precise information of risk arising from specific factors leading to the serious late adverse effects investigated. These potential risk factors (whether treatment or genetic or other) will be made known to those developing new clinical trials, to provide input for future treatment designs that can reduce toxicity while maintaining cure rates. New therapies will be developed that incorporate inter-individual variations in risk, thus ensuring that carefully defined groups of patients receive personalized therapies.

Various target audiences can derive benefit from the information resulting from the project. Apart from the survivors themselves and health care providers, this will allow policy makers to deliver optimal strategies and interventions and to provide better follow-up care. Since childhood cancer late effects may continue life long, the costs to health insurers and public health systems extend far beyond the initial cost of cancer treatment. Risk assessments and implementation of guidelines for care can reduce these costs of cancer survivorship, by both preventing and reducing the incidence of late effects. The installation of advisory boards that have a support function (e.g., on ethical or biostatistical matters), the use of experienced managers, centralized data collection, and the implementation of good communication strategies can provide a template for the organization of international projects using aggregated patient data. One task of good management is to face challenges occurring during the project’s lifetime. This includes taking into account a wide range of interests, dealing with a lack of discipline (e.g., non-production of deliverables within a reasonable timeframe), or with a partner’s refusal to co-operate on elementary issues. As a basic principle, transparent, prompt, and problem-oriented communication, as well as an iron discipline and rigour to reach the objectives, is needed to meet the challenges faced. Further examples of challenges in such complex projects, with a lot of sub-studies and different partners, and how challenges can be met by good project management, are described in the context of another PanCare project [27].

We are leaving behind a carefully-documented legacy of data, design, and methodology that will serve European research well into the future. Actual results from the PanCareLIFE consortium are impressively documented by current publications, e.g., on guidelines and recommendations regarding fertility preservation [28,29,30], the first study on the influence of genetic factors on reduced ovarian function induced by alkylating agents [31], on platinum-induced ototoxicity [32,33,34], health-related quality of life [35], and treatment-related fertility impairment in female survivors [36]. These papers exemplify ways in which PanCareLIFE projects involve researchers and data from across the world.

The consortium will encourage the translation of research results for survivors and their families, as well as for professionals. PanCareLIFE researchers are working with other childhood cancer initiatives to ensure that the value of the data is maximized for future use. The members of the PanCareLIFE consortium will continue to work together following the conclusion of the funded project through a virtual research community established in the closing months of the project. This will also enable the group to complete publication of the results in the future, to complete other publications currently intended, to enable the smooth and efficient archiving of study records and data, and to act as a resource for future researchers.

## 5. Conclusions

Successful delivery of the project aims did not evolve solely from the scientific qualities of the research team. A major impact of PanCareLIFE on European research also lies in the establishment of expertise in managing complex data centrally. Together with the expertise generated within a comparable former project dealing with life threatening late effects (PanCareSurFup), which also established a central point for data collection and harmonization [27], PanCareLIFE has improved the harmonization of data and led to new solutions to data sharing across Europe.

## Figures and Tables

**Table 1:** PanCareLIFE co-operating partners.

Partners	Number of Beneficiaries (in Total 17)	Number of Data Providers (in Total 25)	Members of Management Team (in Total 5)	Members of Other Teams (in Total 6)
**I. Beneficiary, not Data Provider**				
Germany (P. Kaatsch/D. Grabow—Mainz)	1	-	2	-
Ireland (J. Byrne/H. Campbell—Drogheda; K. O’Brien—Dublin)	2	-	3	-
**II. Beneficiary and Data Provider**				
Czech Republic (T. Kepak—Brno; J. Kruesova—Prague)	2	2	-	-
Denmark (J.F. Winther—Copenhagen)	1	1	-	-
France (C. Berger—St. Etienne)	1	1	-	-
Germany (A. Borgmann-Staudt—Berlin; G. Calaminus—Bonn; U. Dirksen—Essen; T. Langer—Lübeck; A. am Zehnhoff-Dinnesen—Münster)	5	5	-	-
Italy (R. Haupt—Genoa)	1	1	-	-
Switzerland (C.E. Kuehni—Bern)	1	1	-	-
The Netherlands (L. Kremer—AMC Amsterdam; E. van Dulmen-den Broeder—VUMC Amsterdam; M.M. van den Heuvel-Eibrink—Rotterdam/Utrecht)	3	3	-	-
**III. Data Provider, not Beneficiary**				
Austria (H. Lackner—Graz)	-	1	-	-
Germany (S. Bielack / S. Hecker-Nolting—Stuttgart; H. Cario—Ulm; M. Kunstreich—Düsseldorf; G. Strauß—Berlin)	-	4	-	-
Israel (D. Modan-Moses—Tel Hashomer)	-	1	-	-
Norway (S. Fossa / E. Ruud —Oslo)	-	1	-	-
Poland (M. Krawczuk-Rybak—Bialystok; J. Stefanowicz—Gdansk)	-	2	-	-
The Netherlands (F. van Leeuwen—Amsterdam)	-	1	-	-
UK (A. Leiper / V. Grandage—London)	-	1	-	-
**IV. Others**				
**Audiological Reference Centre Germany** (A. am Zehnhoff-Dinnesen—Münster)	-	-		1
**Laboratories (Hormone and Genetic Analyses)** Germany (O. Zolk—Ulm) The Netherlands (E. van Dulmen-den Broeder—Amsterdam; A. Uitterlinden / L. Broer / M.M. van den Heuvel-Eibrink—Rotterdam/Utrecht)	-	-		3
**Ethics Advisory Board** Denmark (L.E. Knudsen—Copenhagen)Germany (N.W. Paul—Mainz)	-	-		2

## Data Availability

The data generated in PanCareLIFE will be available after a 5 year embargo following the close of the study (November 2018) and upon request to the chair of the PanCareLIFE Dataset Committee (currently the author D.G.) following the rules set up in the Policy on access to PanCareLIFE Dataset to external researchers.

## Appendix A. Members of the PanCareLIFE Consortium

- PanCareLIFE project partners are: Universitätsmedizin der Johannes Gutenberg-Universität Mainz, Germany (UMC-Mainz: PD Dr. P. Kaatsch, Dr. D. Grabow), Boyne Research Institute, Drogheda, Ireland (Boyne: Dr. J. Byrne, Ms. H. Campbell), Pintail Ltd., Dublin, Ireland (PT: Mr. C. Clissmann, Dr. K. O’Brien), Academisch Medisch Centrum bij de Universiteitvan Amsterdam, Netherlands (AMC: Dr. L.C.M. Kremer), Universität zu Lübeck, Germany (UzL: Professor T. Langer), Stichting VU-VUMC, Amsterdam, Netherlands (VUMC: Dr. E. van Dulmen-den Broeder, Dr. M.H. van den Berg), Erasmus Universitair Medisch Centrum Rotterdam, Netherlands (EMC: Dr. A.C.H. de Vries, Professor M.M. van den Heuvel-Eibrink), Charité - Universitätsmedizin Berlin, Germany (CU: Professor A. Borgmann-Staudt, Mr. R. Schilling), Westfälische Wilhelms-Universität Münster, Germany (UKM: Professor A. am Zehnhoff-Dinnesen), Universität Bern, Switzerland (UNIBE: Professor C.E. Kuehni), Istituto Giannina Gaslini, Genoa, Italy (IGG: Dr. R. Haupt), Fakultni Nemocnice, Brno, Czech Republic (UHB: Dr. T. Kepak), University Hospital, Saint Etienne, France (CHU-SE: Dr. C. Berger), Kraeftens Bekaempelse, Copenhagen, Denmark (KB: Professor J. Falck Winther), Fakultni Nemocnice v Motole, Prague, Czech Republic (FNM: Dr. J. Kruseova), Department of Paediatric Haematology and Oncology, University Hospital Bonn, Germany (UKB: Dr. G. Calaminus, Ms. K Baust) and Prinses Máxima Center for Paediatric Oncology, Utrecht, Netherlands (PMC: Professor M.M. van den Heuvel-Eibrink).
- Data was provided by: Academisch Medisch Centrum bij de Universiteit van Amsterdam, on behalf of the DCOG LATER Study centres, Netherlands (Dr. L.C.M. Kremer), Stichting VU-VUMC, Amsterdam, Netherlands (Dr. E. van Dulmen-den Broeder, Dr. M.H. van den Berg), Erasmus Universitair Medisch Centrum Rotterdam, Netherlands (Dr. A.C.H. de Vries, Professor MM van den Heuvel-Eibrink), Prinses Máxima Centrum (Professor M.M. van den Heuvel-Eibrink), Netherlands Netherlands Cancer Institute (Professor F. van Leeuwen), Charité - Universitätsmedizin Berlin, Germany (Professor A. Borgmann-Staudt, Mr. R. Schilling), Helios Kliniken Berlin-Buch (Dr. G. Strauß), Westfälische Wilhelms-Universität Münster, Germany (Professor A. am Zehnhoff-Dinnesen, Professor U. Dirksen), Universität Bern, Switzerland (Professor C.E. Kuehni), Istituto Giannina Gaslini, Genoa, Italy (Dr. R. Haupt, Dr. M.-L. Garré), Fakultni Nemocnice Brno, Czech Republic (Dr. T. Kepak), University Hospital Saint Etienne, France (Dr. C. Berger), Kraeftens Bekaempelse, Copenhagen, Denmark (Professor J. Falck Winther), Fakultni Nemocnice v Motole, Prague, Czech Republic (Dr. J. Kruseova), Universitetet i Oslo, Norway (Professor S. Fosså), Great Ormond Street Hospital, London, UK (Dr. A. Leiper), Medizinische Universität Graz, Austria (Professor H. Lackner), Medical University of Bialystok, Bialystok, Poland (Dr. A. Panasiuk, Dr. M. Krawczuk-Rybak), Heinrich Heine Universität Düsseldorf, Germany (Dr. M. Kunstreich), Universität Ulm, Germany (Professor H. Cario, Professor O. Zolk), Universität zu Lübeck, Germany (Professor T. Langer), Klinikum Stuttgart, Olgahospital, Stuttgart, Germany (Professor S. Bielack), Uniwersytet Gdánski, Poland (Professor J. Stefanowicz), University College London Hospital, UK (Dr. V. Grandage), Sheba Academic Medical Center Hospital, Ramat Gan, Israel (Professor D. Modan-Moses) and Department of Paediatric Haematology and Oncology, University Hospital Bonn, Germany (Dr. G. Calaminus).
- Independent ethics advice was provided by professors Norbert W. Paul at the University of Mainz, Germany, and Lisbeth E. Knudsen from University of Copenhagen, Denmark.
- Additional PanCareLIFE members served as scientific advisors or had supported formal analyses: Francesca Bagnasco (Epidemiology and Biostatistics Unit, IRCCS Istituto Giannina Gaslini, Genova, Italy), Andrea Bautz and Line Kenborg (Danish Cancer Society Research Center, Copenhagen, Denmark), Jörn D. Beck (Klinik für Kinder und Jugendliche, Friedrich-Alexander- Universität, Erlangen-Nürnberg, Germany), Harald Binder and Daniela Zöller (Institute of Medical Biometry and Statistics, Faculty of Medicine and Medical Center - University of Freiburg, Freiburg, Germany), Linda Broer (Erasmus MC—Sophia Children’s Hospital, Rotterdam, The Netherlands), Leonie Casagranda (Department of Paediatric Hematology and Oncology Unit, University Hospital of Saint-Étienne, Saint-Étienne, France), Eva Clemens (Erasmus MC—Sophia Children’s Hospital, Rotterdam, The Netherlands), Dirk Deuster and Ross Parfitt (Department for Phoniatrics and Pedaudiology, Universitätsklinikum, Münster, Germany), Anna Font-Gonzalez (Department Pediatric Oncology, Emma Children’s Hospital / Academic Medical Center (AMC), Amsterdam, The Netherlands), Stefanie Hecker-Nolting (Department of Paediatrics 5 - Oncology, Hematology and Immunology, Centre for Paediatric, Adolescent and Women’s Medicine, Klinikum Stuttgart—Olgahospital, Stuttgart, Germany), Melanie Kaiser and Claudia Spix (German Childhood Cancer Registry, Division of Childhood Cancer Epidemiology, Institute of Medical Biostatistics, Epidemiology and Informatics (IMBEI), Johannes-Gutenberg University Mainz, Germany), Katerina Kepakova (University Hospital Brno, Brno, Czech Republic), Rahel Kuonen (Swiss Childhood Cancer Registry, Institute of Social and Preventive Medicine, University of Bern, Bern, Switzerland), Erik A. H. Loeffen (Department of Pediatric Oncology/Hematology, University of Groningen, Beatrix Children’s Hospital, University Medical Center Groningen, Groningen, The Netherlands), Ales Luks (Motol Teaching Hospital, Prague, Czech Republic), Gisela Michel (Department of Health Sciences and Medicine, University of Lucerne, Switzerland), Renee Mulder (Department Pediatric Oncology, Emma Children’s Hospital / Academic Medical Center (AMC), Amsterdam, The Netherlands), Andreas Ranft (Pediatrics III, Pediatric Hematology and Oncology, West German Cancer Centre, University Hospital, Essen, Germany), Ellen Ruud (Institutt for klinisk medisin, Universitetet i Oslo, Oslo, Norway)^,^ André G. Uitterlinden (Erasmus MC—Dept. of Internal Medicine, Rotterdam, The Netherlands), Anne-Lotte van der Kooi (Prinses Máxima Center for Paediatric Oncology, Utrecht, The Netherlands), Marloes van Dijk (Amsterdam UMC, Vrije Universiteit Amsterdam, Pediatrics, Cancer Centre Amsterdam, Amsterdam, The Netherlands), Lars Hjorth (Department of Paediatrics, Lund University, Skåne University Hospital, Lund, Sweden).
